# Comprehensive Carrier Screening and Molecular Diagnostic Testing for Recessive Childhood Diseases

**DOI:** 10.1371/4f9877ab8ffa9

**Published:** 2012-05-02

**Authors:** Stephen Kingsmore

## Abstract

Of 7,028 disorders with suspected Mendelian inheritance, 1,139 are recessive and have an established molecular basis. Although individually uncommon, Mendelian diseases collectively account for ~20% of infant mortality and ~18% of pediatric hospitalizations. Molecular diagnostic testing is currently available for only ~300 recessive disorders. Preconception screening, together with genetic counseling of carriers, has resulted in remarkable declines in the incidence of several severe recessive diseases including Tay-Sachs disease and cystic fibrosis. However, extension of preconception screening and molecular diagnostic testing to most recessive disease genes has hitherto been impractical. Recently, we reported a preconception carrier screen / molecular diagnostic test for 448 recessive childhood diseases. The current status of this test is reviewed here. Currently, this reports analytical validity of the comprehensive carrier test. As the clinical validity and clinical utility in the contexts described is ascertained, this article will be updated.

## Clinical Scenarios

The test is designed both for preconception carrier testing of couples wishing to start a family and for molecular diagnosis in children suspected of being affected by a recessive childhood disease. The published (research) version of the test included 448 childhood recessive illnesses with severe clinical manifestations^1^. A revised panel is undergoing clinical validation for use as a laboratory developed test (LDT) with an intention of being offered via a laboratory regulated by the Clinical Laboratory Improvement Amendments (CLIA). The clinical panel contains 595 childhood recessive diseases that are deemed to meet American College of Medical Genetics (ACMG) criteria for implementation of genetic testing for ultra-rare disorders^2^. Validation of analytic utility is being performed for the clinical scenarios detailed below prior to test offering. Initial validation of clinical utility and cost effectiveness will occur over the next year.

1. Preconception carrier testing for recessively inherited diseases of childhood.

Prepregnancy carrier testing is currently offered to couples desiring to start a family in order to provide individualized genetic counseling about risk of conceiving a child affected by a specific recessively inherited diseas^3^ ^4^. The test performs preconception carrier testing for 595 recessive diseases simultaneously and three target populations are envisaged:

i. Couples undergoing *in vitro* fertilization (IVF) procedures. Testing of couples, pretesting of sperm and egg donors and genetic counseling is of utility for reduction in risk of having an affected child. Given the economics of IVF, the incremental cost of carrier testing is unlikely to be a barrier to adoption^5^. Screening of sperm and oocyte donors has lower counseling burden than other clinical scenarios^6^. Further, the motivation of couples undergoing IVF procedures is anticipated to facilitate adoption. Since testing is performed before conception, some of the ethical concerns of carrier testing in other clinical scenarios are not relevant^3^ ^6^. We are not aware of published studies of efficacy in this target population. It should be noted that knowledge of mutations in many of the 595 diseases is incomplete and testing is anticipated to reduce but not eliminate the risk of an affected child.

ii. Individuals and populations at high risk of recessive disorders. Examples include populations with genetic bottlenecks and / or higher rates of consanguinity. Ashkenazi Jewish populations, Arab populations, Amish populations and individuals with a family history of recessive diseases are examples. Preconception testing of motivated populations for recessive disease mutations, together with education and genetic counseling of carriers, can dramatically reduce disease incidence in a generation. The broad rationale is the success of testing north American Ashkenazi Jewish populations for carrier status of Tay-Sachs disease (TSD; Mendelian Inheritance in Man accession number OMIM# 272800)^7^ ^8^ ^9^ ^10^ ^11^ ^12^ ^13^.

iii. General population testing. Given a recent report that we each harbor an average of 2.8 known recessive severe childhood disease mutations^1^, there is theoretical utility of voluntary carrier testing in general populations^14^. The broad rationale is the success of general population testing for carrier status of cystic fibrosis [CF, OMIM#219700]^12^ ^13^ ^15^ ^16^ ^17^ ^18^ ^19^. Practical clinical utility requires a). the cost to be low, b). provision of pre- and post-test genetic counseling (including delineation of the potential benefits and harms of carrier test results) and, c). protections for confidentiality, privacy and against stigmatization or discrimination. The ideal age for recessive disease screening is in early adulthood and before pregnancy. In the US, preconception carrier testing is hospital-based, whereas community-based testing has had success in Canada and Australia^9^ ^19^ ^20^ ^21^ ^22^. Community-based population testing has advantages over testing in a hospital setting, where information about carrier testing often is communicated during pregnancy or after the birth of an affected child^9^ ^19^ ^20^ ^21^ ^22^. Community-based carrier testing has had high uptake, without apparent stigma or discrimination and with substantial reductions in the frequencies of tested disorders^9^ ^19^ ^20^ ^21^ ^22^. Of note, the United Kingdom's Human Genetics Commission recently reported that it found no specific social, ethical or legal principles that would make preconception genetic testing within the framework of a population screening program unacceptable^14^.

Preconception carrier testing for 595 diseases is anticipated to be offered initially as an LDT in late 2011 in the first two clinical scenarios. Expansion to general population testing is anticipated subsequently upon demonstration of cost effectiveness and validation of clinical utility in targeted populations. Revision of national policies for carrier testing is anticipated to be needed in response to next-generation-sequencing based multiplexed tests such as this.

2. Diagnostic testing in potentially affected children.

Diagnostic carrier testing is offered to affected children (via parents) suspected of having a recessively inherited disease in order to determine a definitive diagnosis and, thereby, individualize treatment and genetic counseling^2^. The broad rationale is that the test is an extension of conventional, univariate, serial molecular genetic testing. However, conventional approaches have severe limitations: Hundreds of recessive illnesses exist for which conventional molecular diagnosis is technically feasible but not available. They are too uncommon for commercially-viable conventional genetic testing or blocking patents exist. As a result, knowledge of mutation spectrum, genotype-phenotype relationships and allele frequencies in diseases without molecular diagnosis are rudimentary, inhibiting development of investigational new drugs. Of those for which molecular tests are available, many present as progressive multisystem disorders, requiring lengthy and costly differential diagnosis in a conventional genetic testing scenario, exhausting resources of patients, families and physicians. Thus, typically, <50% of patients undergoing conventional genetic testing receive a molecular diagnosis despite average testing cost per patient of >$10,000. Furthermore, serial univariate testing can take over a year, delaying timely intervention or counseling. It should be noted that our knowledge of mutations in many of the 595 diseases is incomplete and thus testing will not provide definitive diagnosis in all affected children. The scope of diagnostic use of the test is in differential diagnosis of affected children suspected of having one of the 595 diseases. The intended test use is in molecular diagnosis.

## Test Description

The test is as described^1^, but has been modified for clinical testing as follows: Genomic DNA is prepared from patient EDTA-blood samples. 2.6 million nucleotides of target genomic regions, representing exons, intron boundaries and non-exonic mutation containing regions in 527 genes are enriched ~500-fold from the 3.16 billion nucleotide (nt) genome of each sample. Enrichment uses hybrid capture, in which tens of thousands of oligonucleotide probes capture 8,614 genomic DNA fragments, collectively comprising 592 disease genes. Patient DNA is fragmented, denatured and incubated with the oligonucleotides. The target-oligonucleotide hybrids are isolated by magnetic capture^23^. Next generation sequencing of the enriched targets is performed with Illumina HiSeq and TruSeq sequencing-by-synthesis, yielding ~3 billion nucleotides of sequence per sample, each ~125 nucleotides long. Sequences are aligned to the reference human genome uniquely, covering each target nucleotide ~150 times. Alignment uses the algorithm GSNAP^24^ ^25^, with parameters that have been optimized for clinical diagnostic use. Enrichment and sequencing are performed on multiplexed samples, which are disambiguated by molecular barcodes. ~1% of target nucleotides are not covered, while ~95% of target nucleotides have at least 16-fold sequence coverage. The majority of missed nucleotides are in high GC-content targets, are missed reproducibly, and are labelled as such. An automated bioinformatic decision tree is used to identify and genotype variations in the aligned sequences^24^ ^26^ ^27^ ^28^ ^29^ ^30^. Variants are retained if present in at least 8 sequences of quality score >25 and in exons with at least 16-fold sequence coverage^24^. Variants detected in >86% of reads are considered homozygous, while those present in 14-86% of reads are heterozygous. Variants are classified according to ACMG and other guidelines^2^ ^18^ ^31^ ^32^ ^33^ ^34^ ^35^, using literature knowledge as well as *in silico*tools, such as comparison with a variety of mutation and human variation databases, PolyPhen-2 and SIFT, to determine the pathogenicity of each variant. Pathogenic variants are assembled into genotypes and reported. For diagnostic testing, where variants are of uncertain significance, further evidence is sought, using additional *in silico* tools, literature evidence, clinico-pathologic correlation, confirmatory family studies or functional assays, as appropriate. In general, variant interpretation is identical to that performed using conventional molecular diagnostic assays with the exceptions that clinico-pathologic interpretation and masking of non-relevant genes are routine in diagnostic use of the assay and that ~90% of variant annotation and reporting is automated, facilitating interpretation and standardization of reporting. Reporting of variants differs in carrier testing of adults and diagnostic testing of children^31^. Carrier testing reports carrier status in all genes. Diagnostic testing reports positive and negative results in genes relevant to the clinical presentation. Diagnostic testing in children does not report carrier status in genes that are not relevant to presentation^31^. In a subset of cases, further communication between the laboratory director and ordering physician is necessary to guide additional studies and assist in interpretation.

## Public Health Importance

Mendelian diseases collectively affect 13 million people in the US, accounting for ~20% of infant mortality and ~18% of pediatric hospitalizations^36^ ^37^ ^38^ ^39^ ^40^.

Diagnostic testing in potentially affected children

Simultaneous diagnostic testing for 595 recessive childhood diseases is anticipated to have several public health impacts: 1). Extension of the prevention, diagnosis, and treatment benefits demonstrated for conventional genetic testing to hundreds of recessive diseases for which testing is not available today; 2). Reduction in time-to-diagnosis, particularly in illnesses where the differential diagnosis is broad and the conventional approach is serial univariate testing. Serial univariate testing can take over a year, delaying timely intervention or counseling. The initial turnaround time of the test will be 4 weeks. 3). Reduction in cost of diagnosis. The average cost per patient of serial univariate molecular diagnostic testing is ~$10,000 at our institution. The test is anticipated to cost ~$600. 4). Increased rate of definitive molecular diagnosis. Less than 50% of patients undergoing serial univariate molecular diagnostic testing receive a molecular diagnosis. This is anticipated to increase with test use, particularly in illnesses where the differential diagnosis is broad, such as mitochondrial myopathies or intellectual disability. Timely diagnosis of affected individuals has several potential benefits:

1. Prevention of death or markedly diminished disease severity where curative treatments are available. Quite a large number of recessive diseases have specific therapies. Neonatal diagnosis and treatment of phenylketonuria (PKU) and congenital hypothyroidism prevent severe intellectual disability. Likewise, death is prevented in certain forms of congenital adrenal hyperplasia (CAH), medium chain acyl-coA dehydrogenase deficiency (MCAD), and galactosemia (OMIM #230400).

2. Genetic counseling of patients and families about risks for relatives and in additional offspring.

3. Improvement in quality of life in disorders where treatments are ameliorative. While many recessive diseases lack curative treatments, timely diagnosis nevertheless allows specific interventions that can substantially improve quality of life. Such interventions may slow disease progression, lessen symptoms, prevent complications or improve function in affected organ systems.

4. Substantial psychosocial benefits with respect to anxiety, self-image, uncertainty and lifestyle decisions.

5. Multiplexed testing allows rule-out of differential diagnoses, decreasing unnecessary treatments.

Use of the research version of the test revealed that 27% of literature mutations are common polymorphisms or misannotated^1^. Thus, it is critical to establish a clinical grade mutation database for recessive illnesses. Implementation of the test for diagnosis in affected children will, with time, improve the quality and quantity of annotated mutations, particularly for diseases that for which no molecular test is available currently.

In addition, test results have a cumulative potential to inform an understanding of disease mechanisms. In each individual with a Mendelian disorder, the specific mutations impact the age of onset, disease severity, rates of progression, distribution of affected organs, complications, pleiotropy and outcomes. Only in diseases for which molecular diagnosis is undertaken can such knowledge be accumulated. A broad understanding of genotype-phenotype relationships can enable individualized care of patients with recessive diseases. This can potentially include individualized treatment intensity and prediction of disease progression, severity and likely complications. Thus, in the long term, the test, when performed in a research setting, can allow identification of genotype-phenotype relationships that allow conveyance of individualized diagnostic information.

Initial experience with the test has revealed the existence of novel modifier mutations and pleiotropy in patients with recessive illnesses (Kingsmore et al., submitted). Only through multiplexed molecular testing can such knowledge be accumulated. A broad understanding of modifier genes can further enable individualized care of patients with recessive diseases. Thus, in the long term, the test, when performed in a research setting, can allow identification of modifier genes that allow conveyance of individualized diagnostic information.

Finally, timely molecular diagnosis can allow intervention before organ decompensation, when treatment is likely to alter outcomes. Currently, study of new therapies for rare disorders are hampered by diagnosis after organ damage and low rates of ascertainment. Timely diagnosis can permit regional referral of affected individuals for specialized treatment.

It should be noted that substantiation of the potential public health impacts in prevention, diagnosis, or treatment of recessive childhood illnesses is needed. Such assessments should include measurement of cost effectiveness including costs of follow up of ambiguous test results and counseling.

## Published Reviews, Recommendations and Guidelines

Systematic evidence reviews

The emerging use of targeted sequencing of panels of genes, whole exome sequencing and whole genome sequencing for molecular diagnosis of Mendelian diseases was recently reviewed^45^.

Recommendations by independent group

Currently none.

Guidelines by professional groups

The United Kingdom's Human Genetics Commission recently reported guidance on preconception genetic testing within the framework of a population screening program^14^.

## Evidence Overview

Analytic Validity:

The 437 genes responsible for 448 childhood recessive diseases are listed in Table 1. Using genotyping cut-offs of 14% and 86% to differentiate homozygotes and heterozygotes and >20X nucleotide coverage and >10 reads of quality >20 to call a variant, the accuracy of the test for SNP genotyping was 98.8%, analytic sensitivity was 94.9% and analytic specificity was 99.99% for 92,106 SNPs in 26 samples genotyped both by high density arrays and the test^1^. The positive predictive value (PPV) of the test for SNP genotyping was 99.96% and negative predictive value was 98.5%, as ascertained by array hybridization^1^. As sequence depth increased from 0.7 to 2.7GB, test sensitivity increased from 93.9% to 95.6%, whereas PPV remained ~100%. Area under the curve (AUC) of the receiver operating characteristic (ROC) of the test for 92,106 SNP genotypes in 26 samples, when compared with array hybridization, was 0.99 when the number and % reads calling a SNP was varied.

For known substitution, indel, splicing, gross deletion and regulatory alleles in 76 samples, analytic sensitivity was 100% (113 of 113 known alleles). The higher sensitivity for detection of known mutations reflected manual curation. The twenty known indels were confirmed by PCR and Sanger sequencing. Of note, substitutions, indels, splicing mutations and gross deletions account for the vast majority (96%) of annotated mutations^27^.

Unexpectedly, 14 of 113 literature-annotated disease mutations were either incorrect or incomplete. PCR and Sanger sequencing confirmed that the 14 variants and genotypes called by the test were correct^1^.

Gross deletions were detected both by perfect alignment to mutant junction reference sequences and by local decreases in normalized coverage (normalized to total sequence generated). Eleven of eleven gross deletion mutations for which boundaries had been defined were identified^1^. Further analytic validation of ability to detect and genotype gross deletions, gross insertions and complex rearrangements is required.

It should be noted that the clinical version of the test will feature several improvements that are anticipated to improve analytic sensitivity and specificity. These are: 1). Increased depth of sequencing to 3 GB per sample; 2). Automation of the sequencing library preparation and target enrichment; 3). Re-design of the target enrichment oligonucleotides; 4). Change in the variant detection parameters to >16X nucleotide coverage and >6 reads of quality >25 to call a variant; 5). Further refinement of alignment parameters to prevent variant detection solely at the ends of reads; 6). Increased library size to reduce overlap redundancy; 7). Improved sequencing-by-synthesis chemistry (TruSeq); 8). Improved HiSeq instrument specification. Repetition of analytic validation is ongoing in a CLIA-compliant laboratory setting.

Clinical Validity

There are no published systematic evidence reviews of test accuracy, reliability or predictive value in a clinical setting. Experience is being garnered with the use of whole exome or whole genome sequencing for molecular diagnosis of Mendelian diseases and was recently reviewed.

Clinical Utility

There are no published systematic evidence reviews or published clinical trials. Published experience was in a research setting and was not blinded to sample diagnosis^1^. Test development and assessment of analytic and clinical validity and utility are ongoing.

## Links

http://www.beyondbatten.org/

http://www.ncgr.org/preventing-rare-genetic-diseases

http://hematite.ncgr.org/

www.sciencemag.org/content/331/6014/130.full

http://www.npr.org/2011/01/13/132908098/new-gene-test-screens-nearly-500-childhood-diseases

Last updated: *March 18, 2011*

## Table 1

**Table:** 

OMIM#	NAME	GENE
102700	SEVERE COMBINED IMMUNODEFICIENCY, AR, T CELL-NEGATIVE,	ADA
102770	MYOADENYLATE DEAMINASE DEFICIENCY, MYOPATHY DUE TO	AMPD1
105830	ANGELMAN SYNDROME AS	MECP2
107400	PROTEASE INHIBITOR 1; PI	SERPINA1
124000	MITOCHONDRIAL COMPLEX III DEFICIENCY	BCS1L
124000	MITOCHONDRIAL COMPLEX III DEFICIENCY	UQCRB
124000	MITOCHONDRIAL COMPLEX III DEFICIENCY	UQCRQ
133540	COCKAYNE SYNDROME, B; CSB	ERCC6
141800	HEMOGLOBIN--ALPHA LOCUS 1; HBA1	HBA1
141900	HEMOGLOBIN--BETA LOCUS; HBB	HBB
145900	HYPERTROPHIC NEUROPATHY OF DEJERINE-SOTTAS. CMT3, CMT4F	EGR2
145900	HYPERTROPHIC NEUROPATHY OF DEJERINE-SOTTAS. CMT3, CMT4F	MPZ
145900	HYPERTROPHIC NEUROPATHY OF DEJERINE-SOTTAS. CMT3, CMT4F	PMP22
145900	HYPERTROPHIC NEUROPATHY OF DEJERINE-SOTTAS. CMT3, CMT4F	PRX
188055	THROMBOPHILIA DUE TO ACTIVATED PROTEIN C RESISTANCE	F5
190685	DOWN SYNDROME	GATA1
200100	ABETALIPOPROTEINEMIA; ABL	MTTP
200990	ACROCALLOSAL SYNDROME; ACLS	GLI3
201000	CARPENTER SYNDROME	RAB23
201450	ACYL-CoA DEHYDROGENASE, MEDIUM-CHAIN, DEFICIENCY OF	ACADM
201460	ACYL-CoA DEHYDROGENASE, LONG-CHAIN, DEFICIENCY OF	ACADL
201470	ACYL-CoA DEHYDROGENASE, SHORT-CHAIN, DEFICIENCY OF	ACADS
201475	ACYL-CoA DEHYDROGENASE, VERY LONG-CHAIN, DEFICIENCY OF	ACADVL
201710	LIPOID CONGENITAL ADRENAL HYPERPLASIA	CYP11A1
201710	LIPOID CONGENITAL ADRENAL HYPERPLASIA	STAR
201910	CONGENITAL ADRENAL HYPERPLASIA, 21-HYDROXYLASE DEFICIENCY	CYP21A2
202400	AFIBRINOGENEMIA, CONGENITAL	FGA
202400	AFIBRINOGENEMIA, CONGENITAL	FGB
202400	AFIBRINOGENEMIA, CONGENITAL	FGG
203500	ALKAPTONURIA	HGD
203700	ALPERS DIFFUSE CEREBRAL DEGENERATION WITH HEPATIC CIRRHOSIS	POLG
203780	ALPORT SYNDROME, AR	COL4A3
203780	ALPORT SYNDROME, AR	COL4A4
203800	ALSTROM SYNDROME; ALMS	ALMS1
204200	CEROID LIPOFUSCINOSIS, NEURONAL, 3; CLN3	CLN3
204500	CEROID LIPOFUSCINOSIS, NEURONAL, 2; CLN2	TPP1
205100	AMYOTROPHIC LATERAL SCLEROSIS 2, JUVENILE; ALS2	ALS2
206700	ANIRIDIA, CEREBELLAR ATAXIA, AND MENTAL DEFICIENCY	PAX6
207410	ANTLEY-BIXLER SYNDROME; ABS	FGFR2
207900	ARGININOSUCCINIC ACIDURIA	ASL
208000	ARTERIAL CALCIFICATION, GENERALIZED, OF INFANCY; GACI	ENPP1
208085	ARTHROGRYPOSIS, RENAL DYSFUNCTION, AND CHOLESTASIS	VPS33B
208150	FETAL AKINESIA DEATION SEQUENCE; FADS	RAPSN
208400	ASPARTYLGLUCOSAMINURIA	AGA
208540	RENAL-HEPATIC-PANCREATIC DYSPLASIA; RHPD	NPHP3
208900	ATAXIA-TELANGIECTASIA; AT	ATM
208920	EARLY-ONSET ATAXIA WITH OCULOMOTOR APRAXIA AND HYPOALBUMINEMIA	APTX
210210	3-METHYLCROTONYL-CoA CARBOXYLASE 2 DEFICIENCY	MCCC2
210600	SECKEL SYNDROME 1	ATR
210900	BLOOM SYNDROME; BLM	BLM
211600	CHOLESTASIS, PROGRESSIVE FAMILIAL INTRAHEPATIC 1; PFIC1	ATP8B1
211750	C SYNDROME	CD96
212065	CONGENITAL DISORDER OF GLYCOSYLATION, Ia; CDG1A	PMM2
212066	CONGENITAL DISORDER OF GLYCOSYLATION, IIa; CDG2A	MGAT2
212720	MARTSOLF SYNDROME	RAB3GAP2
213700	CEREBROTENDINOUS XANTHOMATOSIS	CYP27A1
214150	CEREBROOCULOFACIOSKELETAL SYNDROME 1; COFS1	ERCC6
214450	GRISCELLI SYNDROME, 1; GS1	MYO5A
214500	CHEDIAK-HIGASHI SYNDROME; CHS	LYST
214950	BILE ACID SYNTHESIS DEFECT, CONGENITAL, 4	AMACR
215045	CHONDRODYSPLASIA, BLOMSTRAND ; BOCD	PTH1R
215100	RHIZOMELIC CHONDRODYSPLASIA PUNCTATA, 1; RCDP1	PEX7
215140	HYDROPS-ECTOPIC CALCIFICATION-MOTH-EATEN SKELETAL DYSPLASIA	LBR
215150	OTOSPONDYLOMEGAEPIPHYSEAL DYSPLASIA; OSMED	COL11A2
215150	OTOSPONDYLOMEGAEPIPHYSEAL DYSPLASIA; OSMED	COL2A1
215600	CIRRHOSIS, FAMILIAL	KRT18
215600	CIRRHOSIS, FAMILIAL	KRT8
215700	CITRULLINEMIA, CLASSIC	ASS1
216400	COCKAYNE SYNDROME, A; CSA	ERCC8
216550	COHEN SYNDROME; COH1	VPS13B
217090	PLASMINOGEN DEFICIENCY, I	PLG
217400	CORNEAL DYSTROPHY AND PERCEPTIVE DEAFNESS	SLC4A11
218000	AGENESIS OF THE CORPUS CALLOSUM WITH PERIPHERAL NEUROPATHY; ACCPN	SLC12A6
219000	FRASER SYNDROME	FRAS1
219000	FRASER SYNDROME	FREM2
219100	CUTIS LAXA, AR, I	EFEMP2
219100	CUTIS LAXA, AR, I	FBLN5
219200	CUTIS LAXA, AR, II	ATP6V0A2
219700	CYSTIC FIBROSIS; CF	CFTR
219750	CYSTINOSIS, ADULT NONNEPHROPATHIC	CTNS
219800	CYSTINOSIS, NEPHROPATHIC; CTNS	CTNS
219900	CYSTINOSIS, LATE-ONSET JUVENILE OR ADOLESCENT NEPHROPATHIC	CTNS
220111	LEIGH SYNDROME, FRENCH-CANADIAN ; LSFC	LRPPRC
220290	DEAFNESS, AR 1A	GJB2
220400	JERVELL AND LANGE-NIELSEN SYNDROME 1; JLNS1	KCNQ1
222448	DONNAI-BARROW SYNDROME	LRP2
222600	DIASTROPHIC DYSPLASIA	SLC26A2
223900	NEUROPATHY, HEREDITARY SENSORY AND AUTONOMIC, III; HSAN3	IKBKAP
224050	CEREBELLAR HYPOPLASIA AND MENTAL RETARDATION	VLDLR
224410	DYSSEGMENTAL DYSPLASIA, SILVERMAN-HANDMAKER ; DDSH	HSPG2
225320	EHLERS-DANLOS SYNDROME, AR, CARDIAC VALVULAR	COL1A2
225410	EHLERS-DANLOS SYNDROME, VII, AR	ADAMTS2
225750	AICARDI-GOUTIERES SYNDROME 1; AGS1	TREX1
225753	PONTOCEREBELLAR HYPOPLASIA 4; PCH4	TSEN54
226600	EPIDERMOLYSIS BULLOSA DYSTROPHICA, AR; RDEB	COL7A1
226650	EPIDERMOLYSIS BULLOSA, JUNCTIONAL, NON-HERLITZ	COL17A1
226650	EPIDERMOLYSIS BULLOSA, JUNCTIONAL, NON-HERLITZ	ITGB4
226650	EPIDERMOLYSIS BULLOSA, JUNCTIONAL, NON-HERLITZ	LAMA3
226650	EPIDERMOLYSIS BULLOSA, JUNCTIONAL, NON-HERLITZ	LAMB3
226650	EPIDERMOLYSIS BULLOSA, JUNCTIONAL, NON-HERLITZ	LAMC2
226670	EPIDERMOLYSIS BULLOSA SIMPLEX WITH MUSCULAR DYSTROPHY	PLEC1
226700	EPIDERMOLYSIS BULLOSA, JUNCTIONAL, HERLITZ	LAMA3
226700	EPIDERMOLYSIS BULLOSA, JUNCTIONAL, HERLITZ	LAMB3
226700	EPIDERMOLYSIS BULLOSA, JUNCTIONAL, HERLITZ	LAMC2
226730	EPIDERMOLYSIS BULLOSA JUNCTIONALIS WITH PYLORIC ATRESIA	ITGA6
226730	EPIDERMOLYSIS BULLOSA JUNCTIONALIS WITH PYLORIC ATRESIA	ITGB4
226980	EPIPHYSEAL DYSPLASIA, MULTIPLE, WITH EARLY-ONSET DIABETES MELLITUS	EIF2AK3
228600	FIBROMATOSIS, JUVENILE HYALINE	ANTXR2
228930	FIBULAR APLASIA OR HYPOPLASIA	WNT7A
229200	BRITTLE CORNEA SYNDROME; BCS	ZNF469
229600	FRUCTOSE INTOLERANCE, HEREDITARY	ALDOB
230000	FUCOSIDOSIS	FUCA1
230400	GALACTOSEMIA	GALT
230500	GM1-GANGLIOSIDOSIS, I	GLB1
230600	GM1-GANGLIOSIDOSIS, II	GLB1
230800	GAUCHER DISEASE, I	GBA
230900	GAUCHER DISEASE, II	GBA
231000	GAUCHER DISEASE, III	GBA
231050	GELEOPHYSIC DYSPLASIA	ADAMTSL2
231530	3-HYDROXYACYL-CoA DEHYDROGENASE DEFICIENCY	HADH
231550	ACHALASIA-ADDISONIANISM-ALACRIMA SYNDROME; AAA	AAAS
231670	GLUTARIC ACIDEMIA I	GCDH
231680	MULTIPLE ACYL-CoA DEHYDROGENASE DEFICIENCY; MADD	ETFA
231680	MULTIPLE ACYL-CoA DEHYDROGENASE DEFICIENCY; MADD	ETFB
231680	MULTIPLE ACYL-CoA DEHYDROGENASE DEFICIENCY; MADD	ETFDH
232200	GLYCOGEN STORAGE DISEASE I	G6PC3
232220	GLYCOGEN STORAGE DISEASE Ib	SLC37A4
232240	GLYCOGEN STORAGE DISEASE Ic	SLC37A4
232300	GLYCOGEN STORAGE DISEASE II	GAA
232400	GLYCOGEN STORAGE DISEASE III	AGL
232500	GLYCOGEN STORAGE DISEASE IV	GBE1
235200	HEMOCHROMATOSIS; HFE	HFE
235200	HEMOCHROMATOSIS; HFE	HFE2
235550	HEPATIC VENOOCCLUSIVE DISEASE WITH IMMUNODEFICIENCY; VODI	SP110
236200	HOMOCYSTINURIA	CBS
236250	HOMOCYSTINURIA DUE TO DEFICIENCY OF METHYLENETETRAHYDROFOLATE	MTHFR
236490	HYALINOSIS, INFANTILE SYSTEMIC	ANTXR2
236670	WALKER-WARBURG SYNDROME; WWS	POMT1
236670	WALKER-WARBURG SYNDROME; WWS	POMT2
236680	HYDROLETHALUS SYNDROME 1	HYLS1
237300	CARBAMOYL PHOSPHATE SYNTHETASE I DEFICIENCY, HYPERAMMONEMIA	CPS1
237310	N-ACETYLGLUTAMATE SYNTHASE DEFICIENCY	NAGS
238970	HYPERORNITHINEMIA-HYPERAMMONEMIA-HOMOCITRULLINURIA SYNDROME	SLC25A15
239000	PAGET DISEASE, JUVENILE	TNFRSF11B
240300	AUTOIMMUNE POLYENDOCRINE SYNDROME, I; APS1	AIRE
241200	BARTTER SYNDROME, ANTENATAL, 2	KCNJ1
241410	HYPOPARATHYROIDISM-RETARDATION-DYSMORPHISM SYNDROME; HRD	TBCE
241510	HYPOPHOSPHATASIA, CHILDHOOD	ALPL
241520	HYPOPHOSPHATEMIC RICKETS, AR	DMP1
241550	HYPOPLASTIC LEFT HEART SYNDROME	GJA1
242300	ICHTHYOSIS, LAMELLAR, 1; LI1	TGM1
242500	ICHTHYOSIS CONGENITA, HARLEQUIN FETUS	ABCA12
242860	IMMUNODEFICIENCY-CENTROMERIC INSTABILITY-FACIAL ANOMALIES SYNDROME	DNMT3B
243500	ISOVALERIC ACIDEMIA; IVA	IVD
243800	JOHANSON-BLIZZARD SYNDROME; JBS	UBR1
244460	KENNY-CAFFEY SYNDROME, 1; KCS	TBCE
245200	KRABBE DISEASE	GALC
245349	PYRUVATE DEHYDROGENASE E3-BINDING PROTEIN DEFICIENCY	PDHX
245400	LACTIC ACIDOSIS, FATAL INFANTILE	SUCLG1
245660	LARYNGOONYCHOCUTANEOUS SYNDROME; LOCS	LAMA3
246200	DONOHUE SYNDROME	INSR
246450	3-HYDROXY-3-METHYLGLUTARYL-CoA LYASE DEFICIENCY	HMGCL
248190	HYPOMAGNESEMIA, RENAL, WITH OCULAR INVOLVEMENT	CLDN19
248500	MANNOSIDOSIS, ALPHA B, LYSOSOMAL	MAN2B1
248600	MAPLE SYRUP URINE DISEASE Ia	BCKDHA
248600	MAPLE SYRUP URINE DISEASE, CLASSIC, IB	BCKDHB
248600	MAPLE SYRUP URINE DISEASE III	DLD
248800	Marinesco-Sjogren Syndrome	SIL1
249000	MECKEL SYNDROME, 1; MKS1	MKS1
249100	FAMILIAL MEDITERRANEAN FEVER; FMF	MEFV
249900	METACHROMATIC LEUKODYSTROPHY DUE TO SAPOSIN B DEFICIENCY	PSAP
250100	METACHROMATIC LEUKODYSTROPHY	ARSA
250250	CARTILAGE-HAIR HYPOPLASIA; CHH	RMRP
250620	BETA-HYDROXYISOBUTYRYL CoA DEACYLASE, DEFICIENCY OF	HIBCH
250950	3-METHYLGLUTACONIC ACIDURIA, I	AUH
251000	METHYLMALONIC ACIDURIA DUE TO METHYLMALONYL-CoA MUTASE DEFICIENCY	MUT
251110	METHYLMALONIC ACIDURIA, cblB	MMAB
251260	NIJMEGEN BREAKAGE SYNDROME	NBN
251880	MITOCHONDRIAL DNA DEPLETION SYNDROME, HEPATOCEREBRAL	C10ORF2
251880	MITOCHONDRIAL DNA DEPLETION SYNDROME, HEPATOCEREBRAL	DGUOK
251880	MITOCHONDRIAL DNA DEPLETION SYNDROME, HEPATOCEREBRAL	MPV17
252150	MOLYBDENUM COFACTOR DEFICIENCY	MOCS1
252150	MOLYBDENUM COFACTOR DEFICIENCY	MOCS2
252500	MUCOLIPIDOSIS II ALPHA/BETA	GNPTAB
252600	MUCOLIPIDOSIS III ALPHA/BETA	GNPTAB
252650	MUCOLIPIDOSIS IV	MCOLN1
252900	MUCOPOLYSACCHARIDOSIS IIIA	SGSH
252930	MUCOPOLYSACCHARIDOSIS IIIC	HGSNAT
253200	MUCOPOLYSACCHARIDOSIS VI	ARSB
253220	MUCOPOLYSACCHARIDOSIS VII	GUSB
253230	MUCOPOLYSACCHARIDOSIS VIII	GNS
253250	MULIBREY NANISM	TRIM37
253260	BIOTINIDASE DEFICIENCY	BTD
253280	MUSCLE-EYE-BRAIN DISEASE; MEB	FKRP
253280	MUSCLE-EYE-BRAIN DISEASE; MEB	POMGNT1
253290	MULTIPLE PTERYGIUM SYNDROME, LETHAL	CHRNA1
253290	MULTIPLE PTERYGIUM SYNDROME, LETHAL	CHRND
253290	MULTIPLE PTERYGIUM SYNDROME, LETHAL	CHRNG
253300	SPINAL MUSCULAR ATROPHY, I; SMA1	SMN1
253310	LETHAL CONGENITAL CONTRACTURE SYNDROME 1; LCCS1	GLE1
253400	SPINAL MUSCULAR ATROPHY, III; SMA3	SMN1
253550	SPINAL MUSCULAR ATROPHY, II; SMA2	SMN1
253800	FUKUYAMA CONGENITAL MUSCULAR DYSTROPHY; FCMD	FKTN
254780	MYOCLONIC EPILEPSY OF LAFORA	EPM2A
254780	MYOCLONIC EPILEPSY OF LAFORA	NHLRC1
254800	MYOCLONIC EPILEPSY OF UNVERRICHT AND LUNDBORG	CSTB
255110	CARNITINE PALMITOYLTRANSFERASE II DEFICIENCY, LATE-ONSET	CPT2
255120	CARNITINE PALMITOYLTRANSFERASE I DEFICIENCY	CPT1A
255960	MYXOMA, INTRACARDIAC	PRKAR1A
256030	NEMALINE MYOPATHY 2; NEM2	NEB
256050	ATELOSTEOGENESIS, II; AOII	SLC26A2
256100	NEPHRONOPHTHISIS 1; NPHP1	NPHP1
256300	NEPHROSIS 1, CONGENITAL, FINNISH ; NPHS1	NPHS1
256370	NEPHROTIC SYNDROME, EARLY-ONSET, WITH DIFFUSE MESANGIAL SCLEROSIS	WT1
256550	NEURAMINIDASE DEFICIENCY	NEU1
256600	NEUROAXONAL DYSTROPHY, INFANTILE; INAD1	PLA2G6
256710	ELEJALDE DISEASE	MYO5A
256730	CEROID LIPOFUSCINOSIS, NEURONAL, 1; CLN1	PPT1
256731	CEROID LIPOFUSCINOSIS, NEURONAL, 5; CLN5	CLN5
256800	INSENSITIVITY TO PAIN, CONGENITAL, WITH ANHIDROSIS; CIPA	NTRK1
256810	NAVAJO NEUROHEPATOPATHY; NN	MPV17
257200	NIEMANN-PICK DISEASE, A	SMPD1
257220	NIEMANN-PICK DISEASE, C1; NPC1	NPC1
257320	LISSENCEPHALY 2; LIS2	RELN
257980	ODONTOONYCHODERMAL DYSPLASIA; OODD	WNT10A
258501	3-METHYLGLUTACONIC ACIDURIA, III	OPA3
259700	OSTEOPETROSIS, AR 1; OPTB1	TCIRG1
259720	OSTEOPETROSIS, AR 5; OPTB5	OSTM1
259730	OSTEOPETROSIS, AR 3; OPTB3	CA2
259770	OSTEOPOROSIS-PSEUDOGLIOMA SYNDROME; OPPG	LRP5
259775	RAINE SYNDROME; RNS	FAM20C
259900	HYPEROXALURIA, PRIMARY, I	AGXT
260000	HYPEROXALURIA, PRIMARY, II	GRHPR
260400	SHWACHMAN-DIAMOND SYNDROME; SDS	SBDS
261515	D-BIFUNCTIONAL PROTEIN DEFICIENCY	HSD17B4
261600	PHENYLKETONURIA; PKU	PAH
261740	GLYCOGEN STORAGE DISEASE OF HEART, LETHAL CONGENITAL	PRKAG2
262300	ACHROMATOPSIA 3; ACHM3	CNGB3
262600	PITUITARY DWARFISM III	HESX1
262600	PITUITARY DWARFISM III	LHX3
262600	PITUITARY DWARFISM III	POU1F1
262600	PITUITARY DWARFISM III	PROP1
263200	POLYCYSTIC KIDNEY DISEASE, AR; ARPKD	PKHD1
263700	PORPHYRIA, CONGENITAL ERYTHROPOIETIC	UROS
264350	PSEUDOHYPOALDOSTERONISM, I, AR; PHA1	SCNN1A
264350	PSEUDOHYPOALDOSTERONISM, I, AR; PHA1	SCNN1B
264350	PSEUDOHYPOALDOSTERONISM, I, AR; PHA1	SCNN1G
264470	PEROXISOMAL ACYL-CoA OXIDASE DEFICIENCY	ACOX1
264700	VITAMIN D-DEPENDENT RICKETS, I	CYP27B1
265000	MULTIPLE PTERYGIUM SYNDROME, ESCOBAR	CHRNG
265100	PULMONARY ALVEOLAR MICROLITHIASIS	SLC34A2
265120	SURFACTANT METABOLISM DYSFUNCTION, PULMONARY, 1; SMDP1	SFTPB
265380	NEWBORN PULMONARY HYPERTENSION, FAMILIAL PERSISTENT	CPS1
265450	PULMONARY VENOOCCLUSIVE DISEASE; PVOD	BMPR2
265800	PYCNODYSOSTOSIS	CTSK
266130	GLUTATHIONE SYNTHETASE DEFICIENCY	GSS
266150	PYRUVATE CARBOXYLASE DEFICIENCY	PC
266200	PYRUVATE KINASE DEFICIENCY OF RED CELLS	PKLR
266265	CONGENITAL DISORDER OF GLYCOSYLATION, IIc; CDG2C	SLC35C1
266900	SENIOR-LOKEN SYNDROME 1; SLSN1	NPHP1
267430	RENAL TUBULAR DYSGENESIS; RTD	ACE
267430	RENAL TUBULAR DYSGENESIS; RTD	AGT
267430	RENAL TUBULAR DYSGENESIS; RTD	AGTR1
267430	RENAL TUBULAR DYSGENESIS; RTD	REN
267450	RESPIRATORY DISTRESS SYNDROME IN PREMATURE INFANTS	SFTPA1
267450	RESPIRATORY DISTRESS SYNDROME IN PREMATURE INFANTS	SFTPB
267450	RESPIRATORY DISTRESS SYNDROME IN PREMATURE INFANTS	SFTPC
268300	ROBERTS SYNDROME; RBS	ESCO2
268800	SANDHOFF DISEASE	HEXB
269250	SCHNECKENBECKEN DYSPLASIA	SLC35D1
269920	INFANTILE SIALIC ACID STORAGE DISORDER	SLC17A5
270200	SJOGREN-LARSSON SYNDROME; SLS	ALDH3A2
270400	SMITH-LEMLI-OPITZ SYNDROME; SLOS	DHCR7
270450	INSULIN-LIKE GROWTH FACTOR I, RESISTANCE TO	IGF1
270550	SPASTIC ATAXIA, CHARLEVOIX-SAGUENAY ; SACS	SACS
271245	INFANTILE-ONSET SPINOCEREBELLAR ATAXIA; IOSCA	C10ORF2
271900	CANAVAN DISEASE	ASPA
271930	STRIATONIGRAL DEGENERATION, INFANTILE; SNDI	NUP62
271980	SUCCINIC SEMIALDEHYDE DEHYDROGENASE DEFICIENCY	ALDH5A1
272300	SULFOCYSTEINURIA	SUOX
272800	TAY-SACHS DISEASE; TSD	HEXA
273395	TETRA-AMELIA, AR	WNT3
274150	THROMBOTIC THROMBOCYTOPENIC PURPURA, CONGENITAL; TTP	ADAMTS13
274270	DIHYDROPYRIMIDINE DEHYDROGENASE; DPYD	DPYD
274600	PENDRED SYNDROME; PDS	SLC26A4
275100	HYPOTHYROIDISM, CONGENITAL, NONGOITROUS, 4; CHNG4	TSHB
275210	TIGHT SKIN CONTRACTURE SYNDROME, LETHAL	LMNA
275210	TIGHT SKIN CONTRACTURE SYNDROME, LETHAL	ZMPSTE24
276700	TYROSINEMIA, I	FAH
276820	ULNA AND FIBULA, ABSENCE OF	WNT7A
276900	USHER SYNDROME, I	MYO7A
276901	USHER SYNDROME, IIA; USH2A	USH2A
276902	USHER SYNDROME, III; USH3	CLRN1
276904	USHER SYNDROME, IC; USH1C	USH1C
277300	SPONDYLOCOSTAL DYSOSTOSIS, AR 1; SCDO1	DLL3
277400	METHYLMALONIC ACIDURIA AND HOMOCYSTINURIA, cblC	MMACHC
277440	VITAMIN D-DEPENDENT RICKETS, II	VDR
277460	VITAMIN E, FAMILIAL ISOLATED DEFICIENCY OF; VED	TTPA
277470	PONTOCEREBELLAR HYPOPLASIA 2A; PCH2A	TSEN54
277580	WAARDENBURG-SHAH SYNDROME	EDN3
277580	WAARDENBURG-SHAH SYNDROME	EDNRB
277580	WAARDENBURG-SHAH SYNDROME	SOX10
277900	WILSON DISEASE	ATP7B
278700	XERODERMA PIGMENTOSUM, COMPLEMENTATION GROUP A; XPA	XPA
278730	XERODERMA PIGMENTOSUM, COMPLEMENTATION GROUP D; XPD	ERCC2
278740	XERODERMA PIGMENTOSUM, COMPLEMENTATION GROUP E	DDB2
278760	XERODERMA PIGMENTOSUM, COMPLEMENTATION GROUP F; XPF	ERCC4
278780	XERODERMA PIGMENTOSUM, COMPLEMENTATION GROUP G; XPG	ERCC5
278800	DE SANCTIS-CACCHIONE SYNDROME	ERCC6
278800	DE SANCTIS-CACCHIONE SYNDROME	XPA
300004	CORPUS CALLOSUM, AGENESIS OF, WITH ABNORMAL GENITALIA	ARX
300018	DOSAGE-SENSITIVE SEX REVERSAL; DSS	NR0B1
300048	INTESTINAL PSEUDOOBSTRUCTION, NEURONAL, CHRONIC IDIOPATHIC, XLR	FLNA
300067	LISSENCEPHALY, XLR, 1; LISX1	DCX
300069	CARDIOMYOPATHY, DILATED, 3A; CMD3A	TAZ
300100	ADRENOLEUKODYSTROPHY; ALD	ABCD1
300209	SIMPSON-GOLABI-BEHMEL SYNDROME, 2	OFD1
300215	LISSENCEPHALY, XLR, 2 LISX2	ARX
300220	MENTAL RETARDATION, XLR, SYNDROMIC 10; MRXS10	HSD17B10
300240	HOYERAAL-HREIDARSSON SYNDROME; HHS	DKC1
300243	MENTAL RETARDATION, XLR, SYNDROMIC, CHRISTIANSON	SLC9A6
300291	ECTODERMAL DYSPLASIA, HYPOHIDROTIC, WITH IMMUNE DEFICIENCY	IKBKG
300301	OSTEOPETROSIS, LYMPHEDEMA, ECTODERMAL DYSPLASIA, ANHIDROSIS, IMMUNODEFICIENCY	IKBKG
300322	LESCH-NYHAN SYNDROME; LNS	HPRT1
300352	CREATINE DEFICIENCY SYNDROME, XLR	SLC6A8
300400	SEVERE COMBINED IMMUNODEFICIENCY, XLR; SCIDX1	IL2RG
300472	AGENESIS OF CORPUS CALLOSUM WITH MENTAL RETARDATION, OCULAR COLOBOMA	IGBP1
300523	ALLAN-HERNDON-DUDLEY SYNDROME AHDS	SLC16A2
300672	EPILEPTIC ENCEPHALOPATHY, EARLY INFANTILE, 2	CDKL5
300673	ENCEPHALOPATHY, NEONATAL SEVERE, DUE TO MECP2 MUTATIONS	MECP2
300755	AGAMMAGLOBULINEMIA, XLR XLA	BTK
301000	WISKOTT-ALDRICH SYNDROME; WAS	WAS
301040	α-THALASSEMIA/MENTAL RETARDATION SYNDROME,NONDELETION , XLR ATRX	ATRX
301500	FABRY DISEASE	GLA
301830	SPINAL MUSCULAR ATROPHY, XLR 2; SMAX2	UBA1
301835	ARTS SYNDROME; ARTS	PRPS1
302045	CARDIOMYOPATHY, DILATED, 3B; CMD3B	DMD
302060	BARTH SYNDROME; BTHS	TAZ
302950	CHONDRODYSPLASIA PUNCTATA 1, XLR RECESSIVE; CDPX1	ARSE
303100	CHOROIDEREMIA; CHM	CHM
303350	MASA SYNDROME	L1CAM
304100	CORPUS CALLOSUM, PARTIAL AGENESIS OF, XLR	L1CAM
304790	IMMUNODYSREGULATION, POLYENDOCRINOPATHY, AND ENTEROPATHY, XLR	FOXP3
305100	ECTODERMAL DYSPLASIA, HYPOHIDROTIC, XLR; XHED	EDA
305900	GLUCOSE-6-PHOSPHATE DEHYDROGENASE; G6PD	G6PD
306955	HETEROTAXY, VISCERAL, 1, XLR; HTX1	ZIC3
307000	HYDROCEPHALUS DUE TO CONGENITAL STENOSIS OF AQUEDUCT OF SYLVIUS; HSAS	L1CAM
308230	IMMUNODEFICIENCY WITH HYPER-IgM, 1; HIGM1	CD40LG
308240	LYMPHOPROLIFERATIVE SYNDROME, XLR, 1; XLP1	SH2D1A
308350	EPILEPTIC ENCEPHALOPATHY, EARLY INFANTILE, 1	ARX
308370	INFERTILE MALE SYNDROME	AR
308930	LEIGH SYNDROME, XLR	PDHA1
309000	LOWE OCULOCEREBRORENAL SYNDROME; OCRL	OCRL
309400	MENKES DISEASE	ATP7A
309500	RENPENNING SYNDROME 1; RENS1	PQBP1
309520	LUJAN-FRYNS SYNDROME	MED12
310200	MUSCULAR DYSTROPHY, DUCHENNE ; DMD	DMD
310400	MYOTUBULAR MYOPATHY 1; MTM1	MTM1
310600	NORRIE DISEASE; ND	NDP
311150	OPTICOACOUSTIC NERVE ATROPHY WITH DEMENTIA	TIMM8A
311250	ORNITHINE TRANSCARBAMYLASE DEFICIENCY, HYPERAMMONEMIA DUE TO	OTC
312060	PROPERDIN DEFICIENCY, XLR	CFP
312080	PELIZAEUS-MERZBACHER DISEASE; PMD	PLP1
312700	RETINOSCHISIS 1, XLR, JUVENILE; RS1	RS1
312750	RETT SYNDROME; RTT	MECP2
312863	COMBINED IMMUNODEFICIENCY, XLR; CIDX	IL2RG
312920	SPASTIC PARAPLEGIA 2, XLR; SPG2	PLP1
314390	VACTERL ASSOCIATION WITH HYDROCEPHALUS, XLR	FANCB
600060	DEAFNESS, NEUROSENSORY, AR 2; DFNB2	MYO7A
600118	WARBURG MICRO SYNDROME; WARBM	RAB3GAP1
600121	RHIZOMELIC CHONDRODYSPLASIA PUNCTATA, 3; RCDP3	AGPS
600143	CEROID LIPOFUSCINOSIS, NEURONAL, 8; CLN8	CLN8
600501	ABCD SYNDROME	EDNRB
600649	CARNITINE PALMITOYLTRANSFERASE II DEFICIENCY, INFANTILE	CPT2
600721	D-2-HYDROXYGLUTARIC ACIDURIA	D2HGDH
600737	INCLUSION BODY MYOPATHY 2, AR; IBM2	GNE
600802	SEVERE COMBINED IMMUNODEFICIENCY, AR, T CELL- B CELL+, NK CELL-	JAK3
600972	ACHONDROGENESIS, IB; ACG1B	SLC26A2
601067	USHER SYNDROME, ID; USH1D	CDH23
601186	MICROPHTHALMIA, SYNDROMIC 9; MCOPS9	STRA6
601378	CRISPONI SYNDROME	CRLF1
601451	NEVO SYNDROME	PLOD1
601457	SEVERE COMBINED IMMUNODEFICIENCY, AR, T CELL-NEGATIVE,	RAG1
601457	SEVERE COMBINED IMMUNODEFICIENCY, AR, T CELL-NEGATIVE,	RAG2
601559	STUVE-WIEDEMANN SYNDROME	LIFR
601675	TRICHOTHIODYSTROPHY, PHOTOSENSITIVE; TTDP	ERCC2
601675	TRICHOTHIODYSTROPHY, PHOTOSENSITIVE; TTDP	ERCC3
601675	TRICHOTHIODYSTROPHY, PHOTOSENSITIVE; TTDP	GTF2H5
601678	BARTTER SYNDROME, ANTENATAL, 1	SLC12A1
601705	T-CELL IMMUNODEFICIENCY, CONGENITAL ALOPECIA, AND NAIL DYSTROPHY	FOXN1
601706	YEMENITE DEAF-BLIND HYPOPIGMENTATION SYNDROME	SOX10
601780	CEROID LIPOFUSCINOSIS, NEURONAL, 6; CLN6	CLN6
601847	CHOLESTASIS, PROGRESSIVE FAMILIAL INTRAHEPATIC 2; PFIC2	ABCB11
602083	USHER SYNDROME, IF; USH1F	PCDH15
602088	NEPHRONOPHTHISIS 2; NPHP2	INVS
602390	HEMOCHROMATOSIS, JUVENILE; JH	HAMP
602390	HEMOCHROMATOSIS, JUVENILE; JH	HFE2
602398	DESMOSTEROLOSIS	DHCR24
602473	ENCEPHALOPATHY, ETHYLMALONIC	ETHE1
602579	CONGENITAL DISORDER OF GLYCOSYLATION, Ib; CDG1B	MPI
602771	RIGID SPINE MUSCULAR DYSTROPHY 1; RSMD1	SEPN1
603147	CONGENITAL DISORDER OF GLYCOSYLATION, Ic; CDG1C	ALG6
603358	GRACILE SYNDROME	BCS1L
603554	OMENN SYNDROME	DCLRE1C
603554	OMENN SYNDROME	RAG1
603554	OMENN SYNDROME	RAG2
603585	CONGENITAL DISORDER OF GLYCOSYLATION, IIf; CDG2F	SLC35A1
603903	SICKLE CELL ANEMIA	HBB
604004	MEGALENCEPHALIC LEUKOENCEPHALOPATHY WITH SUBCORTICAL CYSTS; MLC	MLC1
604250	HEMOCHROMATOSIS, 3	TFR2
604320	SPINAL MUSCULAR ATROPHY, DISTAL, AR, 1; DSMA1	IGHMBP2
604369	SIALURIA, FINNISH	SLC17A5
604377	CARDIOENCEPHALOMYOPATHY, FATAL INFANTILE, DUE TO CYTOCHROME c OXIDASE	SCO2
604498	AMEGAKARYOCYTIC THROMBOCYTOPENIA, CONGENITAL; CAMT	MPL
605039	C-LIKE SYNDROME	CD96
605253	NEUROPATHY, HYPOMYELINATING/CHARCOT-MARIE-TOOTH DISEASE, 4E	EGR2
605253	NEUROPATHY, HYPOMYELINATING/CHARCOT-MARIE-TOOTH DISEASE, 4E	MPZ
605355	NEMALINE MYOPATHY 5; NEM5	TNNT1
605407	SEGAWA SYNDROME, AR	TH
605472	USHER SYNDROME, IIC; USH2C	GPR98
605899	GLYCINE ENCEPHALOPATHY; GCE	AMT
605899	GLYCINE ENCEPHALOPATHY; GCE	GCSH
605899	GLYCINE ENCEPHALOPATHY; GCE	GLDC
606056	CONGENITAL DISORDER OF GLYCOSYLATION, IIb; CDG2B	MOGS
606353	PRIMARY LATERAL SCLEROSIS, JUVENILE; PLSJ	ALS2
606369	EPILEPTIC ENCEPHALOPATHY, LENNOX-GASTAUT	MAPK10
606407	HYPOTONIA-CYSTINURIA SYNDROME	PREPL
606407	HYPOTONIA-CYSTINURIA SYNDROME	SLC3A1
606612	MUSCULAR DYSTROPHY, CONGENITAL, 1C; MDC1C	FKRP
606812	FUMARASE DEFICIENCY	FH
606943	USHER SYNDROME, IG; USH1G	USH1G
606966	NEPHRONOPHTHISIS 4; NPHP4	NPHP4
607014	HURLER SYNDROME	IDUA
607091	CONGENITAL DISORDER OF GLYCOSYLATION, IId; CDG2D	B4GALT1
607095	ANAUXETIC DYSPLASIA	RMRP
607330	LATHOSTEROLOSIS	SC5DL
607426	COENZYME Q10 DEFICIENCY	APTX
607426	COENZYME Q10 DEFICIENCY	CABC1
607426	COENZYME Q10 DEFICIENCY	COQ2
607426	COENZYME Q10 DEFICIENCY	PDSS1
607426	COENZYME Q10 DEFICIENCY	PDSS2
607598	ICOS DEFICIENCY; LCCS2	ERBB3
607616	NIEMANN-PICK DISEASE, B	SMPD1
607624	GRISCELLI SYNDROME, 2; GS2	RAB27A
607625	NIEMANN-PICK DISEASE, C2	NPC2
607626	ICHTHYOSIS, LEUKOCYTE VACUOLES, ALOPECIA, AND SCLEROSING CHOLANGITIS	CLDN1
607655	SKIN FRAGILITY-WOOLLY HAIR SYNDROME	DSP
607855	MUSCULAR DYSTROPHY, CONGENITAL MEROSIN-DEFICIENT, 1A; MDC1A	LAMA2
608013	GAUCHER DISEASE, PERINATAL LETHAL	GBA
608093	CONGENITAL DISORDER OF GLYCOSYLATION, Ij; CDG1J	DPAGT1
608099	MUSCULAR DYSTROPHY, LIMB-GIRDLE, 2D; LGMD2D	SGCA
608456	COLORECTAL ADENOMATOUS POLYPOSIS, AR	MUTYH
608540	CONGENITAL DISORDER OF GLYCOSYLATION, Ik; CDG1K	ALG1
608612	MANDIBULOACRAL DYSPLASIA WITH B LIPODYSTROPHY; MADB	ZMPSTE24
608629	JOUBERT SYNDROME 3; JBTS3	AHI1
608643	AROMATIC L-AMINO ACID DECARBOXYLASE DEFICIENCY	DDC
608688	AICAR TRANSYLASE/IMP CYCLOHYDROLASE, DEFICIENCY OF	ATIC
608782	PYRUVATE DEHYDROGENASE PHOSPHATASE DEFICIENCY	PDP1
608799	CONGENITAL DISORDER OF GLYCOSYLATION, Ie; CDG1E	DPM1
608800	SUDDEN INFANT DEATH WITH DYSGENESIS OF THE TESTES SYNDROME; SIDDT	TSPYL1
608804	LEUKODYSTROPHY, HYPOMYELINATING, 2	GJC2
608836	CARNITINE PALMITOYLTRANSFERASE II DEFICIENCY, LETHAL NEONATAL	CPT2
608840	MUSCULAR DYSTROPHY, CONGENITAL, 1D	LARGE
609015	TRIFUNCTIONAL PROTEIN DEFICIENCY	HADHA
609015	TRIFUNCTIONAL PROTEIN DEFICIENCY	HADHB
609016	LONG-CHAIN 3-HYDROXYACYL-CoA DEHYDROGENASE DEFICIENCY	HADHA
609049	PIERSON SYNDROME	LAMB2
609056	AMISH INFANTILE EPILEPSY SYNDROME	ST3GAL5
609060	COMBINED OXIDATIVE PHOSPHORYLATION DEFICIENCY 1; COXPD1	GFM1
609241	SCHINDLER DISEASE, I	NAGA
609254	SENIOR-LOKEN SYNDROME 5; SLSN5	IQCB1
609304	EPILEPTIC ENCEPHALOPATHY, EARLY INFANTILE, 3	SLC25A22
609311	CHARCOT-MARIE-TOOTH DISEASE, 4H; CMT4H	FGD4
609528	CEREBRAL DYSGENESIS, NEUROPATHY, ICHTHYOSIS, PALMOPLANTAR KERATODERMA	SNAP29
609560	MITOCHONDRIAL DNA DEPLETION SYNDROME, MYOPATHIC	TK2
609583	JOUBERT SYNDROME 4; JBTS4	NPHP1
609638	EPIDERMOLYSIS BULLOSA, LETHAL ACANTHOLYTIC	DSP
610003	CEROID LIPOFUSCINOSIS, NEURONAL, 8, NORTHERN EPILEPSY	CLN8
610006	2-METHYLBUTYRYL-CoA DEHYDROGENASE DEFICIENCY	ACADSB
610090	PYRIDOXAMINE 5-PRIME-PHOSPHATE OXIDASE DEFICIENCY	PNPO
610127	CEROID LIPOFUSCINOSIS, NEURONAL, 10; CLN10	CTSD
610188	JOUBERT SYNDROME 5; JBTS5	CEP290
610198	3-METHYLGLUTACONIC ACIDURIA, V	DNAJC19
610370	DIARRHEA 4, MALABSORPTIVE, CONGENITAL	NEUROG3
610377	MEVALONIC ACIDURIA	MVK
610498	COMBINED OXIDATIVE PHOSPHORYLATION DEFICIENCY 2; COXPD2	MRPS16
610505	COMBINED OXIDATIVE PHOSPHORYLATION DEFICIENCY 3; COXPD3	TSFM
610532	LEUKODYSTROPHY, HYPOMYELINATING, 5	FAM126A
610651	XERODERMA PIGMENTOSUM, COMPLEMENTATION GROUP B; XPB	ERCC3
610688	JOUBERT SYNDROME 6; JBTS6	TMEM67
610725	NEPHROTIC SYNDROME, 3; NPHS3	PLCE1
610768	CONGENITAL DISORDER OF GLYCOSYLATION, Im; CDG1M	DOLK
610854	OSTEOGENESIS IMPERFECTA, IIB	CRTAP
610915	OSTEOGENESIS IMPERFECTA, VIII	LEPRE1
610951	CEROID LIPOFUSCINOSIS, NEURONAL, 7; CLN7	MFSD8
610992	PHOSPHOSERINE AMINOTRANSFERASE DEFICIENCY	PSAT1
611067	SPINAL MUSCULAR ATROPHY, DISTAL, AR, 4; DSMA4	PLEKHG5
611126	ACYL-CoA DEHYDROGENASE FAMILY, MEMBER 9, DEFICIENCY OF	ACAD9
611561	MECKEL SYNDROME, 5; MKS5	RPGRIP1L
611705	MYOPATHY, EARLY-ONSET, WITH FATAL CARDIOMYOPATHY	TTN
611719	COMBINED OXIDATIVE PHOSPHORYLATION DEFICIENCY 5; COXPD5	MRPS22
611721	COMBINED SAPOSIN DEFICIENCY	PSAP
611722	KRABBE DISEASE, ATYPICAL, DUE TO SAPOSIN A DEFICIENCY	PSAP
611726	EPILEPSY, PROGRESSIVE MYOCLONIC 3; EPM3	KCTD7
612164	EPILEPTIC ENCEPHALOPATHY, EARLY INFANTILE, 4	STXBP1
612304	THROMBOPHILIA, HEREDITARY, DUE TO PROTEIN C DEFICIENCY, AUTOSOMAL	PROC
612416	FACTOR XI DEFICIENCY	F11

## Competing Interests

The author has received in-kind funding from private companies (Illumina Inc., Life Technologies Inc., Roche-Nimblegen and British Airways PLC).
